# The Preparation of a Novel Poly(Lactic Acid)-Based Sustained H_2_S Releasing Microsphere for Rheumatoid Arthritis Alleviation

**DOI:** 10.3390/pharmaceutics13050742

**Published:** 2021-05-18

**Authors:** Yue Yu, Zhou Wang, Qian Ding, Xiangbin Yu, Qinyan Yang, Ran Wang, Yudong Fang, Wei Qi, Junyi Liao, Wei Hu, Yizhun Zhu

**Affiliations:** 1 State Key Laboratory of Quality Research in Chinese Medicine & School of Pharmacy, Macau University of Science and Technology, Macau SAR 999078, China; 1709853fct30001@student.must.edu.mo (Y.Y.); 1709853jct30002@student.must.edu.mo (Z.W.); 1909853qct30001@student.must.edu.mo (Q.D.); 1909853ucw30006@student.must.edu.mo (Q.Y.); 1809853pct30001@student.must.edu.mo (R.W.); 1909853scw30001@student.must.edu.mo (Y.F.); wqi@must.edu.mo (W.Q.); 1809853gct20004@student.must.edu.mo (J.L.); 1909853xct30001@student.must.edu.mo (W.H.); 2 School of Pharmacy, Fujian Medical University, Fuzhou 350108, China; yxb4666@fjmu.edu.cn

**Keywords:** S-propargyl-cysteine, poly(lactic acid), endogenous hydrogen sulfide, water-in-oil-in-water, rheumatoid arthritis

## Abstract

Rheumatoid arthritis (RA) is a chronic, inflammatory autoimmune disease that mainly erodes joints and surrounding tissues, and if it is not treated in time, it can cause joint deformities and loss of function. S-propargyl-cysteine (SPRC) is an excellent endogenous hydrogen sulfide donor which can relieve the symptoms of RA through the promotion of H_2_S release via the CSE/H_2_S pathway in vivo. However, the instant release of H_2_S in vivo could potentially limit its further clinical use. To solve this problem, in this study, a SPRC-loaded poly(lactic acid) (PLA) microsphere (SPRC@PLA) was prepared, which could release SPRC in vitro in a sustained manner, and further promote sustained in vivo H_2_S release. Furthermore, its therapeutical effect on RA in rats was also studied. A spherical-like SPRC@PLA was successfully prepared with a diameter of approximately 31.61 μm, yielding rate of 50.66%, loading efficiency of 6.10% and encapsulation efficiency of 52.71%. The SPRC@PLA showed significant prolonged in vitro SPRC release, to 4 days, and additionally, an in vivo H_2_S release around 3 days could also be observed. In addition, a better therapeutical effect and prolonged administration interval toward RA rats was also observed in the SPRC@PLA group.

## 1. Introduction

Rheumatoid arthritis (RA) is a chronic, inflammatory autoimmune disease that mainly erodes joints and surrounding tissues [1,2,3,4] and if it is not treated in time, it can cause joint deformities and loss of function. It is often accompanied by tissue and organ injury, including cardiovascular and lung [5,6,7]. The pathogenesis of RA is still not clear, therefore, an ideal drug has not been found to completely cure this type of disease [8,9]. At present, the treatment of RA is mainly based on non-steroidal anti-inflammatory drugs, glucocorticoids, traditional anti-rheumatic drugs, and biological agents for improving the condition of the disease [10,11,12]. However, the specific treatment of RA is still a dilemma in modern medicine.

Poly(lactic acid) (PLA) is produced by the polymerization of lactic acid. Because of its excellent properties such as good biocompatibility and degradability, it has been widely studied since it was discovered [13,14,15,16]. The initial raw material of PLA is plant starch, which produces extremely low pollution during the production process, and can also be completely decomposed into CO_2_ and H_2_O after use and utilized by nature [17,18]. Due to its excellent performance, PLA has been recognized to be a new type of green and environmentally friendly polymer material. Because of its biodegradability, PLA can be mixed with specific drugs to produce microparticles [19]. When these microparticles reach the action site, they slowly decompose into CO_2_ and H_2_O, and the drug is gradually released at the corresponding action site, therefore, improving the therapeutic index [13,14,20,21].

**Figure 1:**
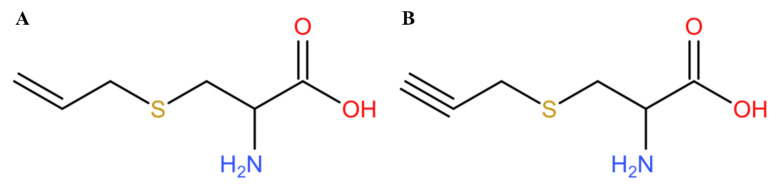
The chemical structure of (**A**) S-allyl-cysteine (SAC) and (**B**) S-propargyl-cysteine (SPRC).

Hydrogen sulfide (H_2_S) has always been considered to be a poisonous gas with a smell similar to rotten eggs, and it is produced in large quantities in some polluted environments [22]. With the deepening of research, recent studies have found that H_2_S is also an important physiological gas molecule and is considered to be the third gasotransmitter after nitric oxide (NO) and carbon monoxide (CO) [23,24]. Endogenous H_2_S is produced via the catalyzation of cystathionine pyridoxal-5-phosphate dependent enzymes, including cystathionine-β-synthase (CBS), cystathionine-y-lyase (CSE), and 3-mercaptopyruvate sulfurtransferase (3-MST). In human blood, the concentration of H_2_S at normal physiological levels is about 40 µM, and the local concentration in the brain can reach more than 100 µM. As a gas signal molecule, H_2_S could reduce high glucose-induced myocardial injury [25] or kidney injury [26], however, the half-life of direct administration of H_2_S is too short, and it is also difficult to precisely control the dosage. Therefore, it is of great significance to study a series of H_2_S donor which could be used as a CSE substrate to further release H_2_S in a relatively slow manner [27,28]. S-allyl-cysteine (SAC) is an extract in garlic, which could reduce the area of myocardial infarction by regulating the level of H_2_S in ischemic myocardial tissue [29,30]. According to the structure of SAC, our group synthesized a compound called S-propargyl-cysteine (SPRC, also known as ZYZ-802) [31,32,33], which is a compound with a similar structure to SAC (Figure 1). Our previous study found that SPRC could be used as a new type of H_2_S donor for ischemia-hypoxic cell models and the treatment of coronary artery ligation rat myocardial infarction models [32,34]. In addition, SPRC could also exert its neuroprotective effect through its anti-inflammatory effect [35,36]. Recent studies have also shown that SPRC could treat rheumatoid arthritis in rats by regulating endogenous H_2_S [31,37,38]. However, the instant release of H_2_S by SPRC might prevent its clinical use, hence, how to achieve a sustained release of H_2_S in vivo through SPRC remains to be a challenging problem.

In this study, we aim to solve the problem that SPRC might prompt the H_2_S in an instant manner, a SPRC-loaded PLA-based microsphere was successfully prepared (SPRC@PLA), which showed sustained release of SPRC in vitro, therefore, elevating the plasma H_2_S concentration for almost 3 days. Through this long elevation period, the administration interval for treating RA has also been increased as compared with that determined in a previous study [31].

## 2. Materials and Methods

### 2.1. Materials

Poly(lactic acid) (PLA) polymer, with Mw around 10,000~18,000 Da and viscosity of 0.16~0.24 was purchased from Evonik Industries (AG, Essen, Germany). SPRC was synthesized, as previously reported [39,40]. Poly(vinyl alcohol) (PVA), with MW around 25,000 Da, and 88% mole hydrolyzed, was purchased from Polysciences (Warrington, PA, USA). Elisa kit of TNF-α, IL-1β, IL-6, and IL-10 were purchased from MultiSciences (Hangzhou, Zhejiang, China). Complete Freund’s adjuvant (CFA), monobromobimane (MBB), diethylenetriaminepentaacetic acid (DTPA), dichloromethane (DCM), and acetonitrile were purchased from Macklin Industrial Corporation (Shanghai, China).

### 2.2. The Preparation of SPRC-Loaded Poly(Lactic Acid) (PLA) Microsphere (SPRC@PLA)

A double emulsion evaporation method (W1/O/W2) was followed with slight modification [41]. First, 50 mg of SPRC was dissolved in 1 mL of distilled water to prepare the inner water phase (W1). Meanwhile, the oil phase (O), which was various amounts of PLA dissolved in 12 mL of DCM, was also prepared. Then, the W1 was dispersed in O, with further emulsification through an Ultraturrax T25 high-speed homogenizer (IKA, Staufen, Germany) at 9000~12,000 rpm for 5 min to prepare the primary water-in-oil emulsion (W1/O). Then, the W1/O was dispersed in 100 mL of 0.5% (*w*/*w*) PVA solution with a paddle agitation at 800 rpm for 4 h, until the evaporation of organic solvent. Then, particles were collected through filtration via sieve with 200 mesh, intended to remove the potential bulk shape microspheres, and washed three times with distilled water to remove the excess residual PVA and SPRC on the surface. Finally, the obtained particles were lyophilized overnight to obtain the SPRC@PLA. Table 1 showed the detailed information of the different formulations.

### 2.3. The Production Yeild of SPRC@PLA

The percentage of production yield (PY) was calculated using the following equation:

PY (%) = (W_SPRC@PLA_)/(W_PLA_ + W_SPRC_), (1)

where W is the weight of corresponding component.

### 2.4. The Quantification of SPRC

The loaded drug (SPRC) was quantified by HPLC method, as reported with little modification [42]. Briefly, an Agilent 1200 series HPLC system (Santa Clara, CA, USA) was used to detect SPRC samples from physicochemical properties. A reversed-phase HPLC column (Agilent C18 column, 250 mm × 4.6 mm, 5 μm) was used. The mobile phase was chosen as acetonitrile and water. The detection wavelength was set as 220 nm. The gradient procedure was as follows: 0–1 min, 3% acetonitrile; 1–2 min, 3–15% acetonitrile; 2–5 min, 15–25% acetonitrile; 5–7 min, 25% acetonitrile; 7–10 min, 25–30% acetonitrile The wavelength of SPRC was determined as 220 nm, the flow rate was 0.5 mL·min^−1^, the column temperature was set as 35 °C, and the sample injection volume was 20 μL.

### 2.5. The Morphology Study of SPRC@PLA

Samples were firstly dispersed in distilled water, and a Microtrac S3500 (Montgomeryville, PA, USA) was used for the measurement of particle size and size distribution. A Phenom Pro Desktop SEMS-3400 scanning electron microscope (Thermo Fisher Scientific Inc., Waltham, MA, USA) was conducted to observe the morphology of SPRC@PLA. Samples were gold coated before examination.

### 2.6. The Encapsulation Efficiency of SPRC@PLA

The loading efficiency (LE) and encapsulation efficiency (EE) were determined by dissolving 50 mg of SPRC@PLA in 3 mL of DCM with further extract SPRC with 5 mL distilled water and analyzed using a 1290 Infinity II LC System (Agilent Technologies, Inc., Santa Clara, CA, USA). LE and EE were calculated using the following equations:

LE (%) = (W_SPRCi_ − W_SPRCs_)/W_SPRC@PLA_ (2)

EE (%) = (W_SPRCi_ − W_SPRCs_)/W_SPRCi_ (3)

where W_SPRCi_ is the weight of SPRC initially fed, W_SPRCs_ is the weight of SPRC in supernatant, and W_SPRC@PLA_ is the weight of SPRC@PLA.

### 2.7. The SPRC Release In Vitro

The in vitro releasing experiment was conducted, using the method as reported with little modification [43]. First, 50 mg of differently prepared SPRC@PLA was dispersed in vials filled with 3 mL of PBS buffer (pH = 7.4, 37 °C) and placed in a shaker bath with a constant shaking speed of 100 rpm and temperature at 37 °C (Clifton Shaking Bath NE5, Nikel Electro Ltd., Weston-super-Mare, UK). Then, 0.5 mL of the samples were taken out, and then the same volume of PBS was refilled at predetermined intervals, and samples were analyzed using a 1290 Infinity II LC System (Agilent Technologies Inc., Santa Clara, CA, USA).

### 2.8. The Measurement of H_2_S Release In Vivo

**Figure 2:**
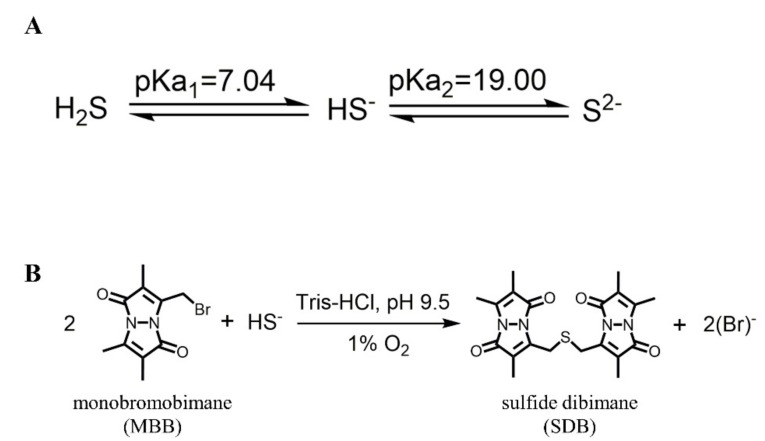
The mechanism for the detection of H_2_S. (**A**) The acid dissociation constant of H_2_S; (**B**) the mechanism of monobromobimane (MBB) reaction with HS^−^ to produce SDB in an alkaline and hypoxia environment.

The concentration of H_2_S was measured, as reported with little modification [44], and the schematic for detection of H_2_S in vivo is shown in Figure 2. Briefly, 15 μL of serum sample, 25 μL of MBB acetonitrile solution, and 35 μL of 0.3% DTPA containing Tris-HCl buffer (pH 9.5) were mixed and incubated in a hypoxia incubator for 30 min. Subsequently, 25 μL of sulfosalicylic acid was added to stop the reaction, and then centrifugated at 12,000 rpm for 10 min. Finally, 30 μL of supernatant, 267 μL of acetonitrile, and 3 μL of internal standard (hydrocortisone methanol solution) were mixed and analyzed with LC-MS.

Samples were analyzed using an Agilent 1200 series HPLC system (Agilent Technologies Inc., Santa Clara, CA, USA) coupled with an Agilent 6460 Triple Quadrupole (Agilent Technologies Inc., Santa Clara, CA, USA). A ZORBAX Eclipse Plus 95 C18, 2.1 × 50 mm, 1.8 μm column (Agilent Technologies Inc., Santa Clara, CA, USA) was used and temperature was set at 35 °C. The mobile phase consisted of water (A) and acetonitrile (B) and the gradient delivery was as follows: at 0–0.5 min, 5% B; 0.5–0.6 min, 5–20% B; 0.6–5.0 min, 20–47.5% B; 5.0–5.1 min, 47.5–95% B; 5.1–6.0 min, 95% B, at a flow rate of 0.3 mL·min^−1^. The mass spectrometer was operated in positive ion mode. The scan type chosen was MRM with gas temperature at 325 °C and gas flow at 10 L·min^−1^. Scan time was 500 ms and start-stop mass was 100~1000. The sample injection volume was 5 μL.

### 2.9. The SPRC@PLA Promoted H_2_S Release In Vivo

The Animal Care and Use Committee of Municipal Affairs Bureau of Macau approved all studies described herein (approval number AL010/DICV/SIS/2018, 23 June 2018), and the experiment was conducted under the guidance of the *NIH Guide for the Care and Use of Laboratory Animals* (8th edition). The neonatal Sprague-Dawley (SD) rats were purchased from the University of Hong Kong.

Samples of SPRC powder and SPRC@PLA were dissolved or dispersed in saline for subcutaneous injection, each sample contained the same amount of SPRC, and the amounts used were calculated through the weight of the rats (100 mg·kg^−1^). The rats’ serum was collected at predetermined times (0, 0.5, 1, 1.5, 2, 3, 6, 12, 24, 48, and 72 h) into heparin sodium tubes and analyzed. Each group contained 3 rats.

### 2.10. SPRC@PLA Showed Anti-Inflmmation Effect towards Rheumatoid Arthritis

The AIA rat model was established via the injection of CFA (10 mg·mL^−1^), according to the manufacturer’s instructions. In total, 30 rats were randomly divided into four groups as follows: Control group (*n* = 5), no intervention; AIA group (*n* = 5), injection of 100 μL of CFA; SPRC group (*n* = 5), after injection of 100 μL of CFA, further subcutaneous injected with 2 mL of SPRC solution every 3 days for 30 days; SPRC@PLA group (*n* = 5), after injection of 100 μL of CFA, further subcutaneous injected with 2 mL of SPRC@PLA suspension every 3 days for 30 days (the amounts of SPRC used were all equivalent to 100 mg·kg^−1^ of SPRC.)

The paw volume was measured using a UGO Basile 7140 plethysmometer (Ugo Basile, Gemonio VA, Italy) and body weight was measured at the 0, 5th, 15th, 20th, 25th, and 30th day post the injection of CFA. The arthritis index was scored (Table 2) from 0 to 4 per limb, with 0 = no sign of inflammation and 1~4 = increasing degrees of inflammation, and a maximum score of 16 per rat.

At day 30, a blood sample was collected from rats in each group, the pro-inflammatory cytokine levels (TNF-α, IL-1β, and IL-6) and anti-inflammatory cytokine (IL-10) level in serum were measured using ELISA kits, according to the manufacturer’s instructions.

### 2.11. Statistical Analysis

Statistical analyses of samples were performed using IBM SPSS Statistics Base (V22, IBM, Armonk, NY, USA.) and GraphPad Prism (V8, GraphPad Software, San Diego, CA, USA). Each experiment was performed at least three times. The data are expressed as the mean ± SD. Statistical significance was determined using a one-way analysis of variance (ANOVA) test, unless otherwise stated, *p* < 0.05 was considered to be significant.

## 3. Results

### 3.1. The Characterization of SPRC@PLA

First, the influence of PLA was investigated. As shown in Table 3, with an increase in the amount of PLA used, a decreasing trend of LE and an increasing trend of EE could be observed. The influence of the amount of PLA used on particle size was also investigated. The particle size showed an increasing trend with an increase in the amount of PLA used. While interestingly, there is no significant influence on PY.

The influence of homogenization speed was also investigated. As shown in Table 4, with an increase in homogenization speed, the LE and EE both showed an increasing trend while conversely, the particle size showed a decreasing trend. Interestingly, the PY still showed no significant change with an increase in homogenization speed.

**Figure 3:**
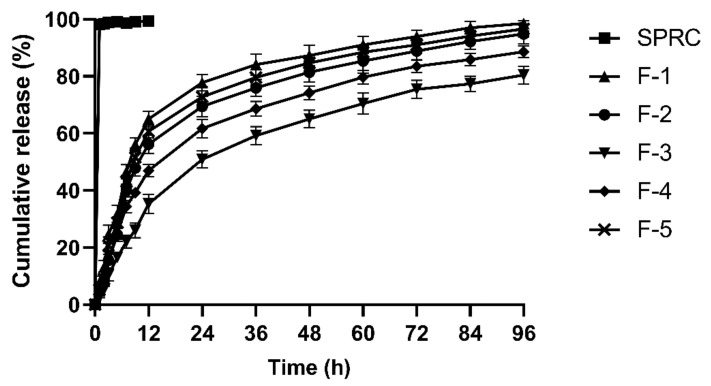
The in vitro release of SPRC from SPRC@PLA in PBS (*n* = 3, mean ± SD).

The in vitro release profiles from F-1 to F-5 were also investigated for the selection of the optimized formulations, and the results are shown in Figure 3. SPRC might dissolve extremely fast in PBS due to its high hydrophilicity. However, a significantly prolonged in vitro release period could be observed in F-1 to F-5 as compared with the SPRC group, which was up to almost 4 days. F-3 and F-4 both showed an incomplete cumulative release potential due to the larger size usually accompanied with a relatively sustained release manner. In addition, F-1, F-2, and F-5 all showed a sustained and complete release within 96 h. By combining the in vitro release results with the particle size, it could be deduced that particle size might play a vital role in the property of in vitro release.

F-1 to F-5 showed a similar PY, which indicated that neither the amount of PLA used, nor the homogenization speed could influence the PY of the prepared SPRC@PLA. Normally, for subcutaneous injection, micro-sized particles with a range between 20 to 100 μm [45] are generally used, since, on the one hand, it usually has sufficient size to incorporate enough active pharmaceutical ingredient, and, on the other hand, sizes between this range are normally suitable, and therefore do not induce inflammation in the injection area. Hence, although F-1 and F-5 both showed a sustained and complete release of SPRC, for further in vivo study, F-1 and F-5 were not selected.

**Figure 4:**
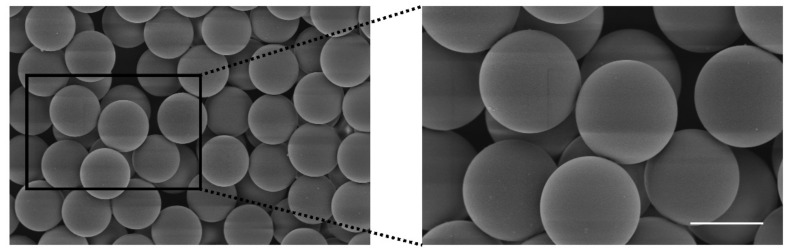
The SEM of SPRC@PLA microspheres (scale bar = 20 μm).

Above all, F-2 was chosen as the optimized formulation for further study due to its high EE, stable PY, relative monodispersed particle size, as well as its sustained and complete release in vitro. The SEM of F-2 is shown in Figure 4. For a convenient expression, F-2 is denoted as SPRC@PLA for the remainder of this paper.

### 3.2. The Elevation of Plasma H_2_S Concentration by Supplementations

**Figure 5:**
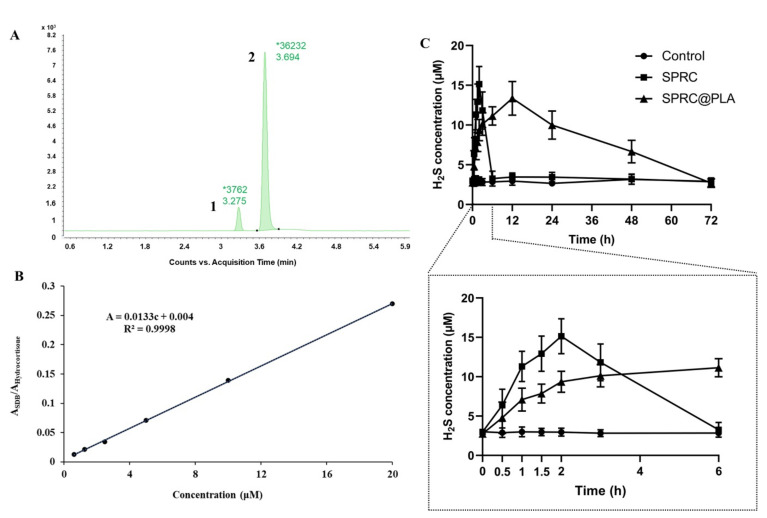
The detection of endogenous H_2_S via LC-MS. (**A**) The chromatography of SDB, peak 1/SDB, peak 2/hydrocortisone (internal standard); (**B**) the calibration curve of SDB in different concentration (0.625~20 μM); (**C**) the 3-day endogenous H_2_S concentration changes in plasma after a single injection of SPRC or SPRC@PLA. Dosage of 100 mg kg^−1^ was calculated according to rats’ body weight (*n* = 3, mean ± SD).

Generally, H_2_S is unstable, and can exist as the mixed state of hydrogen sulfide (H_2_S), hydrogen sulfide anion (HS^−^), and sulfide anion (S^2−^) under physiological conditions (Figure 1A). It has been reported [46,47] that H_2_S, HS^−^, or S^2−^ could react quickly with MBB to produce a relatively stable SDB in a Tris-HCl buffer (pH 9.5) under 1% oxygen (Figure 1B). It would be much easier to detect the SDB rather than the H_2_S in vivo. Herein, the LC-MS was adopted for the measurement of SDB in vivo, which could indirectly reflect the equal amount of H_2_S in vivo. Two peaks, as shown in Figure 5A, indicated effective separation of SDB (Peak 1) and internal standard (Peak 2). Then, the calibration curve was calculated with a concentration range from 0.625 to 20 μM, (Figure 5B) which indicated a good linear correlation of this method. The SPR promoted H_2_S release in vivo in a fast manner, while SPRC@PLA sustained the elevated plasma H_2_S concentration, as shown in Figure 5C. Instantaneous H_2_S production and metabolism could be observed within 6 h after a single injection of SPRC solution. Differently, SPRC@PLA slowly elevated the plasma H_2_S concentration, which was potentially induced by the sustained SPRC release from SPRC@PLA.

### 3.3. Supplementations Increased the Expression of CSE

**Figure 6:**
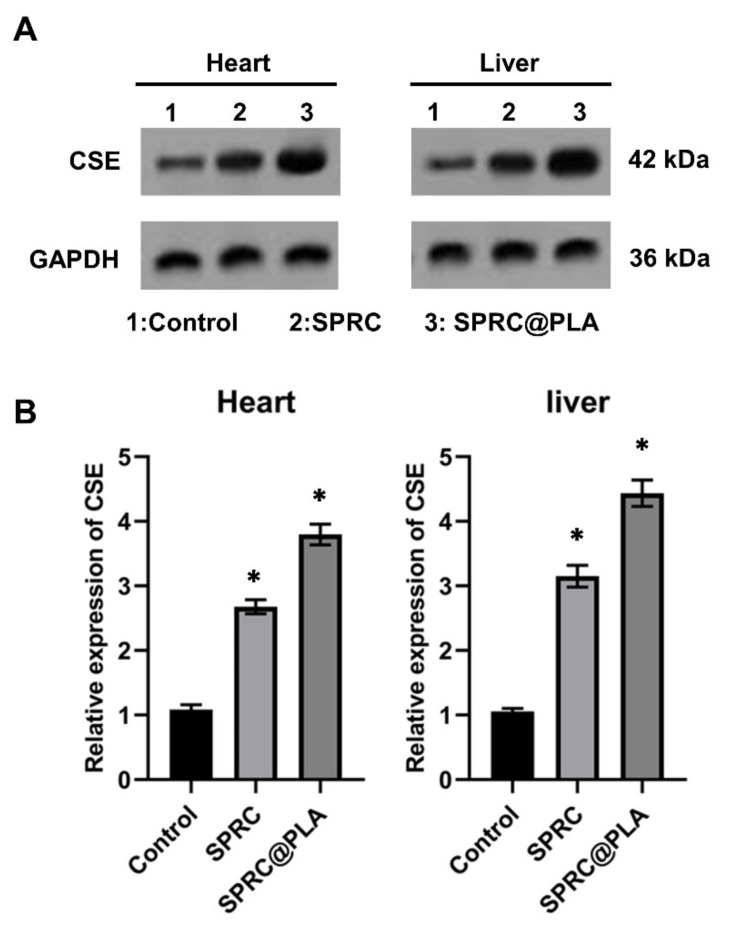
**(A)** The expression of CSE in hearts and liver of rats determined by Western blot and (**B**) the relative expression fold changes. GAPDH was used as a loading control and (*) indicated significant different as compared with the control group (*n* = 5, mean ± SD).

The SPRC has been reported to be able to promote the H_2_S release in vivo through the CSE/H_2_S signaling pathway, according to our previous studies [6,7], and the CSE mainly distributed in heart and liver [48,49,50,51]. The expression of CSE in the heart and liver were investigated and the increased expression could be found in both SPRC and SPRC@PLA groups. The SPRC@PLA group showed a higher expression of CSE than the SPRC group both in heart and liver, mainly because of the sustained release of SPRC from SPRC@PLA (Figure 6).

### 3.4. Supplementations Inhibited the Paw Swollen in AIA Rats

**Figure 7:**
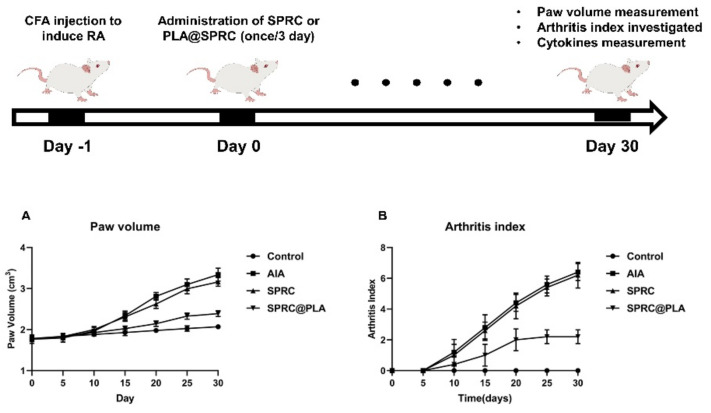
The (**A**) paw volume was measured by plethysmometer, and the (**B**) arthritis index were assessed using the arthritis scoring system to evaluate the severity of swollen symptoms (*n* = 5, mean ± SD).

As shown in Figure 7, the paw swollen was calculated through the paw volume and arthritis index, while before the 10th day, no significant increase of these parameters could be observed. However, after 10 days, an instant and dramatic increase of paw volume and arthritis index could be observed in the AIA model group, while the SPRC group showed the same trend, which indicated a low therapeutical effect of the SPRC group. Conversely, the SPRC@PLA group showed significant inhibition of both paw volume and arthritis index.

**Figure 8:**
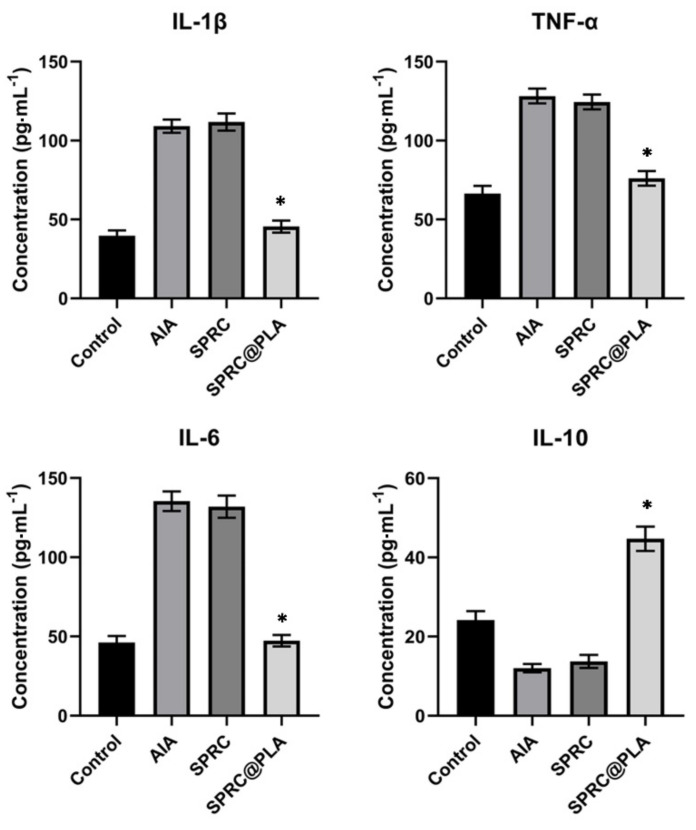
The pro-inflammatory cytokine levels of IL-1β, TNF-α, and IL-6, and anti-inflammatory cytokine level of IL-10 in rats were measured. * indicated a significant different as compared with the AIA model group (*n* = 5, mean ± SD).

As illustrated in Figure 8, SPRC showed a negligible anti-inflammatory effect in AIA rats as compared with the model group, while a dramatic decrease in pro-inflammatory cytokines and an increase in anti-inflammatory cytokines could be observed in the SPRC@PLA group.

## 4. Conclusions

In summary, in this study, SPRC@PLA, a spherical-like microsphere, with a diameter of approximately 30 μm was successfully prepared via the W1/O/W2 emulsification method. In addition, SPRC@PLA showed sustained in vitro SPRC release up to 4 days, and this prolonged in vitro release also promoted in vivo H_2_S release in a sustained manner for 3 days with a single injection. In addition, a once per three-day injection of SPRC@PLA showed good therapeutical effect towards AIA, which increased the administration intervals as compared with those in our previous study [31].

## Figures and Tables

**Table 1:** The formulations of different SPRC-loaded poly (lactic acid) (PLA) microspheres (SPRC@PLAs).

Formulations	SPRC	W1	PLA	DCM	HS	W2
F-1	50 mg	1 mL	400 mg	12 mL	12,000 rpm	100 mL
F-2	50 mg	1 mL	800 mg	12 mL	12,000 rpm	100 mL
F-3	50 mg	1 mL	1200 mg	12 mL	12,000 rpm	100 mL
F-4	50 mg	1 mL	800 mg	12 mL	9000 rpm	100 mL
F-5	50 mg	1 mL	800 mg	12 mL	15,000 rpm	100 mL

W1 is the distilled water volume, HS is the homogenization speed, and W2 is the PVA volume.

**Table 2:** The arthritis scoring system.

Arthritis Score	Degree of Inflammation
0	No erythema and swelling
1	Erythema and mild swelling confined to the tarsals or ankle joint
2	Erythema and mild swelling extending from the ankle to the tarsals
3	Erythema and moderate swelling extending from the ankle to metatarsal joints
4	Erythema and severe swelling encompassing the ankle, foot, and digits; ankylosis of the limb might be present

**Table 3:** The influence of the amount of PLA used (*n* = 3, mean ± SD).

Samples	PLA	PY	LE	EE	Particle Size
F-1	400 mg	(49.81 ± 0.61)%	(10.14 ± 0.57)%	(44.94 ± 2.34)%	(13.28 ± 1.90) μm
F-2	800 mg	(50.66 ± 0.55)%	(6.10 ± 0.27)%	(52.71 ± 2.16)%	(31.61 ± 2.01) μm
F-3	1200 mg	(49.45 ± 0.55)%	(4.44 ± 0.20)%	(55.04 ± 2.19)%	(51.60 ± 2.07) μm

PY, production yield; LE, loading efficiency; EE, encapsulation efficiency.

**Table 4:** The influence of homogenization speed (*n* = 3, mean ± SD).

Samples	HS	PY	LE	EE	Particle Size
F-4	9000 rpm	(50.18 ± 0.68)%	(5.25 ± 0.36)%	(45.05 ± 3.45)%	(47.78 ± 2.84) μm
F-2	12,000 rpm	(50.66 ± 0.55)%	(6.10 ± 0.27)%	(52.71 ± 2.16)%	(31.61 ± 2.01) μm
F-5	15,000 rpm	(49.79 ± 0.69)%	(7.05 ± 0.20)%	(57.52 ± 2.54)%	(20.39 ± 2.72) μm

Where the HS: homogenization speed; the PY: production yield; LE: loading efficiency; EE: encapsulation efficiency.

## Data Availability

Data can be received from the authors upon reasonable request.
